# Efficacy of systemic administration of riboflavin on a rabbit model of corneal alkali burn

**DOI:** 10.1038/s41598-020-74484-0

**Published:** 2020-10-14

**Authors:** Maksym Żuk, Ekaterina Lobashova, Olga Żuk, Sławomir Wierzba

**Affiliations:** 1 Faculty of Health Sciences, University of Opole, Katowicka 68, Opole, Poland; 2 Department of Pharmacology, Odessa National Medical University, Valikhovskiy lane, 2., Odessa, Ukraine; 3 Institute of Environmental Engineering and Biotechnology, University of Opole, Kominka 6 A, Opole, Poland

**Keywords:** Medical research, Outcomes research

## Abstract

Changes in the barrier mechanisms in the eye should determine the rational route for the administration and dosage of each drug in the treatment of traumatic injuries and other pathologies. The aim of this study was to examine the efficacy of intra-arterial delivery of ^14^C-riboflavin (as an “indicator”) and compare it with intravenous and intramuscular administration in an animal model of chemical eye burn. ^14^C-riboflavin (^14^C-I) was administered by intra-arterial (carotid artery), intravenous (femoral vein) and intramuscular (femoral muscle) routes. The total radioactivity was determined over 2 h in the plasma and structures of the rabbit’s eyes using a scintillation counter. The results of the study show that intravascular administration of ^14^C-I gives significantly higher concentrations of total radioactivity in the blood and is accompanied by a significant increase in the permeability of the blood-barrier and barrier in eyes suffering from burns. The highest concentration in the plasma and aqueous humour of the anterior chamber of the eye was observed during the first hour with the intra-arterial route of administration of ^14^C-I in either burnt and unburnt eyes. The distribution of total radioactivity in the structures of the eye over the 2 h of the experiment showed a higher level of the drug under intra-arterial administered in the uveal regions, namely: the iris, ciliary body, choroid, retina and also the sclera and cornea. This experimental model shows that intra-arterial administration can increase the bioavailability of a drug to the structures of the eye within a short period of time.

## Introduction

Ocular chemical burns are a significant problem and are not only a local process, therefore the treatment applied should be complex. This applies to the use of systemic antibiotic therapy to reduce the risk of wound infection, the administration of pain relief, and the control and correction of inflammation in the acute phase with drugs^1, 2^.

Chemical damage to the eye, especially alkali burns that quickly penetrate the structures of the eye, is a real problem and requires immediate evaluation and treatment. In this regard, the assessment of the most effective route for drug delivery in the shortest possible time is very important. Ophthalmic pharmacokinetics, although based on the fundamental concept of pharmacokinetics, has its own characteristics associated with the barrier properties created by the tissues of the eye and the routes of drug administration to the eyes^3–7^.

Until now, the question remains unanswered as to which method of drug delivery is the most effective and ensures the supply of therapeutic doses of the drug to the eye tissues^8–10^.

The least effective route of administration is through enteral preparations, due to the relatively slow absorption of the active substance into the bloodstream from the gastrointestinal tract and the first-pass effect through the liver^11^. Instillation into conjunctiva, and intravenous administration are characterized by rapid penetration to the eye structures, but, in practice, does not create sufficient therapeutic concentration^12, 13^. Thus, systemic administration of many drugs, particularly large, or hydrophilic, molecular drugs and their delivery to the posterior segment of the eye, is very limited^14, 15^. Low molecular weight and lipophilic drugs can penetrate the barriers, achieving significant concentrations in the retina and vitreous tissue after systemic administration^16, 17^. However, this effect is levelled by a large blood volume of distribution. This creates the need for large doses of drugs when they are administered systemically, rather than by local injection in order to obtain a sufficient concentration gradient in the choroid and retina.

When alkali gets into the eye, it damages not only the tissues directly at the point of contact, but is able to penetrate into the deeperg parts of the eye, and can cause damage to the choroid and retina. In this case, intravitreal drug delivery is a very successful method of delivery^18, 19^. However, intravitreal administration is fraught with serious complications such as retinal detachment, hemophthalmos, endophthalmitis, sterile endophthalmitis, secondary glaucoma, and cataracts^14, 19–21^.

By Panahi et al.^22^ it was shown that a high intravitreal concentration of non-steroidal anti-inflammatory drugs in the damaged eye can also be achieved with oral administration, which is a more convenient and less traumatic method.

A promising direction in methods of delivering drugs to different parts of the eye in the case of burns can be delivery through contact lenses with drugs. This allows for a long-term effect on the damaged eye^23^. The search for other possible drug carriers continues^24^.

Thus, the effective treatment of ophthalmic diseases is a challenging task for scientists and clinicians^21, 24, 25^.

This study investigates the efficacy of intra-arterial delivery of ^14^C-riboflavin (chosen as a "tracer") and compares it with systemic administration approaches: intravenous and intramuscular administration in an animal model of ocular chemical burn.

## Materials and methods

### Chemicals

This study used labelled ^14^C-riboflavin (^14^C-I) with a specific activity of mCi mmol^−1^ (Amersham, USA). In our work riboflavin was used as a model water soluble drug and was introduced in an isotonic solution of NaCl.

### Animals

The experiments were conducted on 18 Chinchilla breed Rabbits, of both sexes, weighing 2–2.5 kg. The animals were obtained from the breeding facility of the Odessa National Medical University (Odessa, Ukraine)*.* Before the experiment, all the rabbits were assessed for corneal abnormalities and were confirmed to have normal corneas. The investigation was conducted according to the ARVO Statement on the Use of Animals in Ophthalmic and Vision Research and the experimental protocols were approved by the Ethics Committee of the Odessa National Medical University (approval no. 65B/2016).

### Experimental procedure

The 18 rabbits were randomly divided into three groups. In each animal one eye was kept as a control (unburnt), the second for the burn. The rabbits were anaesthetised with a combination of intramuscular ketamine hydrochloride (50 mg kg^−1^) and xylazine hydrochloride (5 mg kg^−1^). The right corneas of the anesthetized rabbits were injured by dropping 10% sodium hydroxide (NaOH) on the corneal surface (0.1 ml over 10 s. alkali injuring the whole cornea, including the limbal region). Then the eyes were immediately rinsed with an excess of 0.9% saline solution. After inducing the chemical burn, the animals were administered ^14^C-riboflavin in the volume of 0.2 ml (2.5 10^6^ dpm kg^−1^): Groups: I—intra-arterially (carotid artery), II—intravenously (femoral vein), III—intramuscularly (the femoral muscle).

Aqueous humour samples and blood plasma from the femoral vein were collected for 2 h at intervals of 10 min. After this, the rabbits were euthanized with intravenous Phenobarbitone sodium (Sigma) (125 mg kg^−1^). This was followed by dissection of both the burnt and healthy eyes. Total radioactivity was determined in biological tissue samples using a Tri Carb 2700 liquid scintillation counter (Canberra Packard, USA).

### Statistical and Pharmacokinetic analysis

Calculation of total radioactivity in tissues after the injection of ^14^C-I (mean ± SEM) was performed using Microsoft Excel. WinNonlin 5.3. (Pharsight, St. Louis, MO, USA) was used to conduct the following parameter analyses: maximal plasma concentration (*C*_*max*_), time to maximal plasma concentration (*T*_*max*_), area under the plasma concentration–time curve from time 0 to t and to infinite (*AUC*_*0-inf*_), absorption rate constant (*k*_*a*_), absorption half-life (*t*_*1/2a*_), elimination rate constant (*k*_*e*_), and elimination half-life (*t*_*1/2e*_). Mean Residence Time (MRT) was calculated using the moment theory method (AUMC/AUC).

Calculation of total area under the first-moment curve (plot of *C*_*t*_ vs *t*) was performed by combining a trapezoid calculation of *AUMC*_*(0-t)*_ and extrapolated area Total *AUC* and *AUMC* computed using exponential terms.

Statistical analysis was performed using the Student's t test and AVOVA, with *p* ≤ 0.05 as the minimal level of significance.

## Results

### Distribution of ^14^C-riboflavin in the rabbit plasma and aqueous humour in healthy and burnt eyes

**Figure 1:**
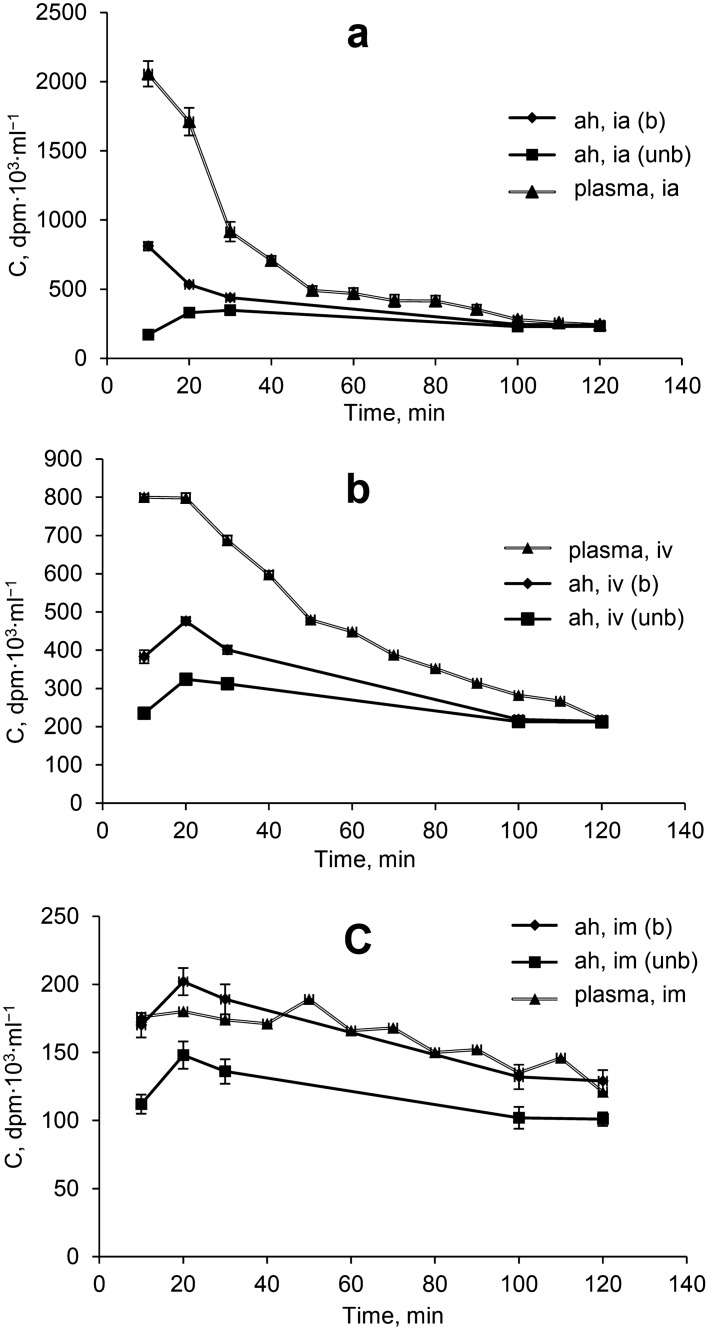
Distribution of the total radioactive material (C, dpm 10^3^ ml^−1^) in plasma and the aqueous humour of anterior chamber in unburnt and burnt eyes after intra–arterial (**a**), intravenous (**b**) and intramuscular (**c**) routes of administration of ^14^C-I. Abbreviations: ah—aqueous humour, (unb)—unburnt eye, (b)—burnt eye.

The investigation of the distribution of ^14^C-I in the rabbit blood under different routes of administration (Fig. 1) demonstrated the significant influence of the route of administration on the pharmacokinetic parameters of the drug. The highest total radioactivity in plasma was observed with intra-arterial administration of riboflavin in the initial study period (10–60 min). The smallest radioactive counts were found in plasma after intramuscular administration in the research time. The process of plasma distribution in the intra-arterial administration is characterized by a two-phase process: α-phase was observed within 10 to 60 min of the experiment, β-phase was from 60 to 120 min.

We noted that for intra-arterial injection of ^14^C-I after 10 min, total radioactivity in plasma was 4 times higher than after intravenous injection and about 16 times higher than with intramuscular administration (Table 1). The results show that in the β-phase monoexponential decline of the elimination rate is predominant for all routes of administration.

**Table 1:** Pharmacokinetic parameters of the distribution of ^14^C-I in plasma of rabbits after intra-arterial, intravenous and intramuscular administration.

Parameters	Intra-arterial administration	Intravenous administration	Intramuscular administration
*k*_*e*_ (min^−1^) (α-phase)	0.0375 ± 0.007		
*k*_*e*_ (min^−1^) (β-phase)	0.0119 ± 0.002	0.0121 ± 0.0001	0.003 ± 0.0001
*t*_*1/2*_ (h)	0.97 ± 0.094	0.95 ± 0.06	3.61 ± 0.30***
*C*_max_. (dpm 10^3^ ml^−1^)	2057 ± 92.2	800 ± 23.4***	180 ± 24.7***
*T*_max_. (min)	10	10	20
*C*_*o*_ ( dpm 10^3^ ml^−1^) (α-phase)	3148.9 ± 198.5		
*C*_*o*_ (dpm 10^3^ ml^−1^) (β-phase)	875.6 ± 85.3	945.3 ± 19.1	195.8 ± 9.5***
*AUC*_*0-t*_ ((dpm 10^3^ ml^−1^) min)	81,975 ± 593.3	55,230 ± 109.7***	19,595 ± 187.63***
*MRT* (h) (β-phase)	1.21 ± 0.04	1.40 ± 0.07	5.15 ± 0.19***

*p < 0.05; **p < 0.01; ***p < 0.001.

As shown in Table 1, the pharmacokinetics of ^14^C-I in the α-phase with intra-arterial administration are characterized by higher values (*p* ≤ 0.001) for rate constants of elimination by plasma, but in the β-phase with intravascular injection this parameter is identical. Characteristic for the drug pharmacokinetics in the blood after the intramuscular route of administration are low levels of total radioactivity (≈ 1.5–2 times lower than that of intravascular administration) and the slowest rate of its elimination (an observed steady-state level of concentration over the entire range of the experiment). This result in a longer time required for the elimination of the drug from the body and accordingly extends its retention time–the half-life is about 4 h, and MRT equals ~ 5 h.

Research of absorption and distribution of ^14^C-I in the aqueous humour of the anterior chamber using intravascular and intramuscular administration shows (Fig. 1) fast delivery of the drug in the aqueous humour of the anterior chamber of healthy and burnt eyes. As can be seen from Fig. 1, after the absorption phase a monoexponential decline in drug concentration is observed in the central (blood) and peripheral (aqueous humour) compartment for all routes of administration.

Intra-arterial administration is characterized by high speed entry into the peripheral compartment (burnt eyes) (Table 2).

**Table 2:** Kinetic parameters of the distribution of total radioactivity in anterior chamber fluid in intact and burnt eyes after intra-arterial administration of ^14^C-riboflavin.

Parameters	Alkali—burned eye	Unburnt (control) eye
*k*_ab_ (min^−1^)	–	0.0355 ± 0.006
*k*_e_ (min^−1^) (α-phase)	0.042 ± 0.008	–
*k*_e_ (min^−1^) (β-phase)	0.008 ± 0.001	0.004 ± 0.0005
*t*_1/2_ (h)	2.08 ± 0.14	2.62 ± 0.86
*C*_max_ (dpm 10^3^ ml^−1^)	811 ± 22.4	348 ± 13.4***
*T*_max_ (min)	10	30
*C*_o_ (dpm 10^3^ ml^−1^)	588.8 ± 28.5	398.1 ± 19.1***
*AUC*_0-t_ ((dpm 10^3^ ml^−1^) min)	44,435 ± 393.3	31,600 ± 109.7***
*MRT* (h)	2.1 ± 0.04	4.2 ± 0.17**

*p < 0.05; **p < 0.01; ***p < 0.001.

The maximum level of total radioactivity after 20 min is observed in all the investigated objects, except burnt eyes, when administered intra-arterially, wherein the maximum concentration is already observed in the first interval of the monitoring—10 min (Tables 2, 3, 4).

**Table 3:** Kinetic parameters of the distribution of total radioactivity in anterior chamber fluid in intact and burnt eyes after intravenous administration of ^14^C-riboflavin.

Parameters	Alkali—burned eye	Unburt (control) eye
*k*_ab_ (min^−1^)	0.0217 ± 0.01	0.0321 ± 0.006
*k*_e_ (min^−1^) (β-phase)	0.008 ± 0.001	0.005 ± 0.0005***
*t*_1/2_ (h)	2.06 ± 0.18	2.51 ± 0.30**
*C*_max_ (dpm 10^3^ ml^−1^)	476 ± 12.1	324 ± 15.4***
*T*_max_ (min)	20	20
*C*_o_ (dpm 10^3^ ml^−1^)	532.4 ± 22.32	354.9 ± 19.3**
*AUC*_0-t_ ((dpm 10^3^ ml^−1^) min)	36,625 ± 435.3	29,920 ± 158.3***
*MRT* (h)	2.2 ± 0.31	4.2 ± 0.12**

*p < 0.05; **p < 0.01; ***p < 0.001.

**Table 4:** Kinetic parameters of the distribution of total radioactivity in anterior chamber fluid in intact and burnt eyes after intramuscular administration of ^14^C-riboflavin.

Parameters	Alkali—burned eye	Unburnt (control) eye
*k*_ab_ (min^−1^)	0.0172 ± 0.006	0.0279 ± 0.008
*k*_e_ (min^−1^) (β-phase)	0.006 ± 0.0004	0.004 ± 0.0005*
*t*_1/2_ (h)	2.73 ± 0.28	2.96 ± 0.37
*C*_max_ (dpm 10^3^ ml^−1^)	202 ± 8.92	148 ± 14.5**
*T*_max_ (min)	20	20
*C*_o_ (dpm 10^3^ ml^−1^)	218.8 ± 13.20	155.9 ± 8.54**
*AUC*_0-t_ ((dpm 10^3^ ml^−1^) min)	18,510 ± 265.7	13,640 ± 178.1***
*MRT* (h)	2.9 ± 0.21	4.1 ± 0.32*

*p < 0.05; **p < 0.01; ***p < 0.001.

This suggests that the α-phase ends during the first interval measurements of concentrations (10–20 min) and a further parallel (in semi-logarithmic coordinates) decrease in concentration in the central and one of the peripheral compartments is the result of the implementation of the β-phase (elimination phase) ^14^C-I and its metabolites.

The rate of absorption of ^14^C-I differed significantly only for intramuscular administration to burnt eyes. Injection of the preparation into the carotid artery was the most effective route of administration for the rapid achievement of the maximum concentration of the drug for unburnt and burnt eyes. The *AUC*_*tissue*_*/AUC*_*pl*_ ratio over time was linear, with intravenous and intramuscular administration of ^14^C-I, after intra-arterial administration a non-linear process was observed in the first hour of the experiment (α-phase). The constants of elimination from the aqueous humour of the unburnt eyes had no significant differences (Tables 2, 3, 4). Alkaline burns significantly alter the permeability of the blood barrier—ANOVA revealed significant differences in the values of total radioactivity (*AUC*) in the anterior chamber of burnt eyes using all routes of administration of ^14^C-I. The burn led to a significant effect on the concentration rate in the aqueous humour of burnt eyes—the elimination rate was in practice twice as high as that for the unburnt eye. Estimation of the distribution kinetics parameters after alkaline burns to the eye shows the characteristics of the process: the increase in the extensive ophthalmopharmacokinetic parameters—i.e. "maximum concentration" and "area under the concentration–time curve" (Tables 2, 3, 4).

Comparative analysis of the level of total radioactivity in anterior chamber fluid with intra-arterial and intravenous administration demonstrated a significantly higher level of the drug in the alkali burnt eye when injected into the carotid artery.

### Contents of ^14^C-I in the structures of burnt and unburnt rabbit eyes

**Figure 2:**
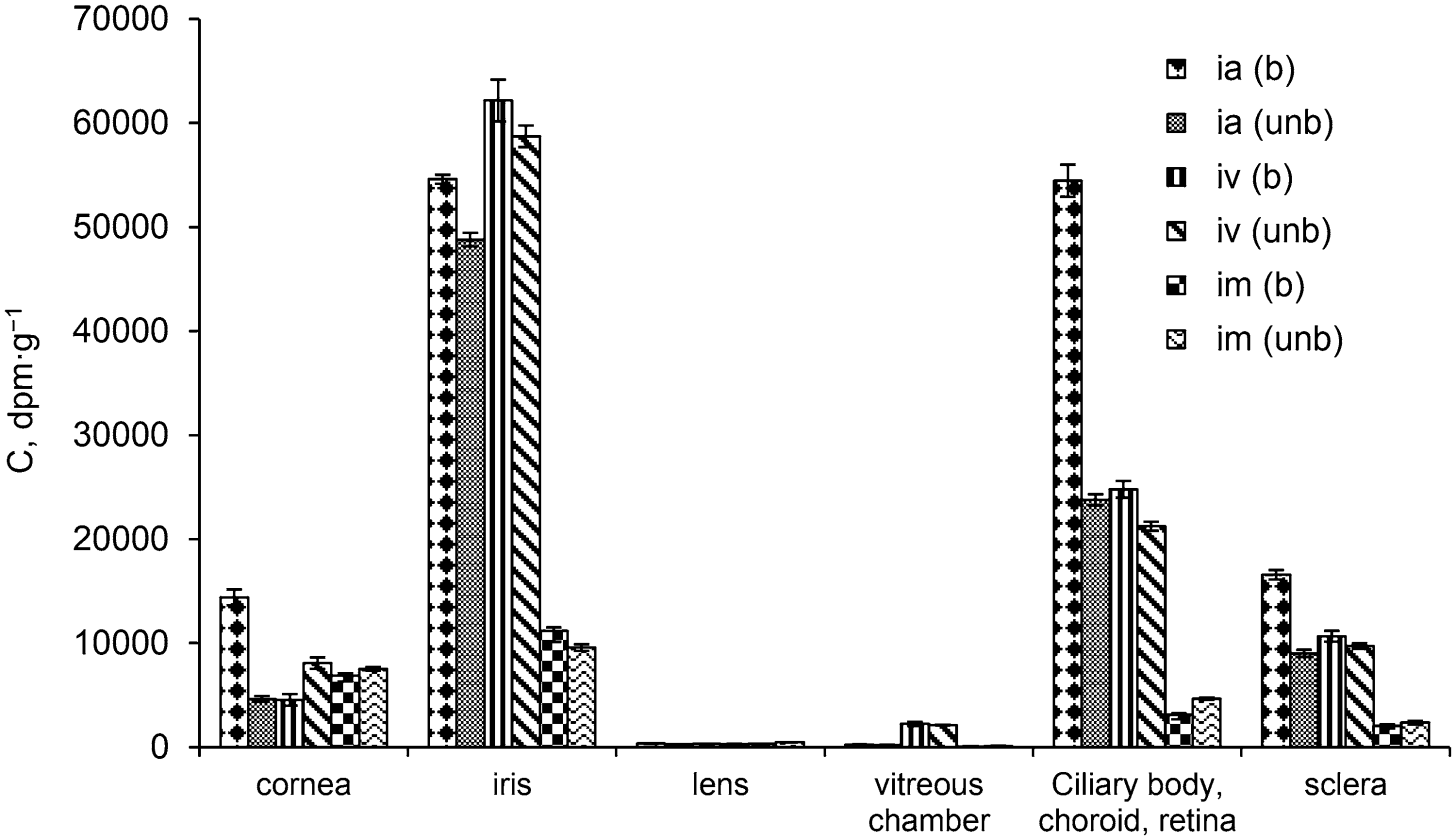
Contents of ^14^C-I in the structures of rabbit eye after intra-arterial, intravenous and intramuscular administration. Abbreviations: (ia)—intra-arterial administration; (iv)—intravenous administration; (im)—intramuscular administration; (unb)—unburnt eye, (b)—burnt eye.

Our pharmacokinetic measurements demonstrate that using all schemes of its administration ^14^C-I and its radioactive metabolites are found in all investigated tissues of a healthy eye (Fig. 2). In different routes, intravascular administration of riboflavin was significantly larger than it was when administered intramuscularly in all tissues of the eye supplied with blood vessels, namely: the iris, ciliary body, choroid, retina and the sclera and the vitreous body.

Regardless of the route of administration, the absorption process was characterized by the same regularities—the content of total radioactivity in the tissues of the eye increased in the following order: vitreous body < lens < cornea < sclera < ciliary body < iris.

Experimental alkali burns to rabbit eyes cause a change in the distribution of radioactive riboflavin in different parts of the rabbit eye and this was dependent on the manner of drug administration (Fig. 2). The equally high concentrations of ^14^C-I found in the iris, ciliary body, sclera and cornea of burnt and unburnt eyes with intravenous administration appears to be explained by the significant permeability of the blood-barrier toward riboflavin (Fig. 2).

After intra-arterial administration of ^14^C-riboflavin, the same regularities in the distribution of the study drug were detected, as was the case after intravenous injection (Fig. 2). Significant accumulation of total radioactivity was observed in the iris, ciliary body, sclera and cornea. After 2 h, the total content of radioactive material in these structures of aburnt eye is more than twice that of other means of administration. Burns caused a significant (*p* < 0.0067) (i.e. 3-fold) increase in the concentration of ^14^C- riboflavin in the cornea and an almost 2-fold increase in the sclera.

Using intramuscular administration, a significantly lower drug concentration was found in all investigated structures of burnt and unburnt eyes than after intravascular administration.

## Discussion

During the first 60 min, the route of administration significantly affected the levels of riboflavin in plasma and the aqueous humour of the anterior chamber of the rabbits’ eyes. Our studies demonstrated that, in the initial period, the highest total radioactivity levels were noted in the investigated tissues under intra-arterial administration of riboflavin. Intra-arterial (instantaneous) administration of drugs can increase the effectiveness of pharmaceuticals effects, as a result of “first pass effect" through perfused organ (tissue) of whole doses. After being administered intravenously, it is carried out “first pass" through the administered dose of the pulmonary circulation, which can reduce the effect of the drug. The availability of the first pass effect through the organ and the absence of it through pulmonary circulation assumes a large pharmacotherapy efficacy of intra-arterial administration of drugs.

In early studies of the effect of eye burns on blood supply, a pronounced decrease in blood supply to the vascular tract in the first hours after injury was noted. In our study, the pharmacokinetic parameters of changes in the drug content in blood plasma during the studied time period demonstrated the absence of this phenomenon.

Intra-arterial administration of drugs can be used for other eye injuries/illnesses. This is evidenced by the work of Kucherenko et al^26, 27^ where it was shown that prolonged infusion of a mixture of drugs through the superficial temporal artery in patients with various eye diseases (neuritis, endophthalmitis, and burns) was a highly effective method of treatment. It has also been shown that, although intravenous administration of drugs provides stable retinoblastoma control, intraarterial chemotherapy has been shown to be most effective for retinoblastoma^22, 28–30^.

Experimental burns cause an increase in the vascular permeability of the endothelium of the eye, which leads to an increase in the drug concentrations in almost all the tested sections of the eye, especially in the iris, ciliary body and retina with the choroid, sclera. It should be noted that with intra-arterial administration, a fairly high concentration of the drug is observed in the burnt cornea, which is the first to undergo changes under chemical action. The intravascular route for the administration of ^14^C-I allows for a significant increase in the content of the drug in blood, as well as in the structures of the eye.

## Conclusion

These results, taken together with the pharmacokinetic properties of ^14^C-I, provide an opportunity to offer long-term use of intra-arterial administration of the drug at a constant rate as a perspective method of administration. This route of administration gives an advantage due to the introduction of the first pass effect through the organ (eyes) throughout the range of pharmacotherapeutic effects. The slow and steady-state rate of elimination of the drug from the plasma and the structures of the eye when administered intramuscularly enables the creation of a fixed concentration. This experimental model shows that intra-arterial administration can increase the bioavailability of a drug to the structures of the eye within a short period of time, which is necessary to provide an intense and effective therapeutic effect.
